# Effects of Humic Acid and Suspended Solids on the Removal of Heavy Metals from Water by Adsorption onto Granular Activated Carbon

**DOI:** 10.3390/ijerph120910475

**Published:** 2015-08-27

**Authors:** Danious P. Sounthararajah, Paripurnanda Loganathan, Jaya Kandasamy, Saravanamuthu Vigneswaran

**Affiliations:** Faculty of Engineering and Information Technology, University of Technology, Sydney, NSW 2007, Australia; E-Mails: Danious.P.Sounthararajah@student.uts.edu.au (D.P.S.); Paripurnanda.Loganathan@uts.edu.au (P.L.); jaya.kandasamy@uts.edu.au (J.K.)

**Keywords:** adsorption, granular activated carbon, heavy metals, humic acid, dissolved organic carbon

## Abstract

Heavy metals constitute some of the most dangerous pollutants of water, as they are toxic to humans, animals, and aquatic organisms. These metals are considered to be of major public health concern and, therefore, need to be removed. Adsorption is a common physico-chemical process used to remove heavy metals. Dissolved organic carbon (DOC) and suspended solids (SS) are associated pollutants in water systems that can interact with heavy metals during the treatment process. The interactions of DOC and SS during the removal of heavy metals by granular activated carbon were investigated in batch and fixed-bed column experiments. Batch adsorption studies indicated that Langmuir adsorption maxima for Pb, Cu, Zn, Cd, and Ni at pH 6.5 were 11.9, 11.8, 3.3, 2.0, and 1.8 mg/g, respectively. With the addition of humic acid (HA) (DOC representative), they were 7.5, 3.7, 3.2, 1.6, and 2.5 mg/g, respectively. In the column experiment, no breakthrough (complete removal) was obtained for Pb and Cu, but adding HA provided a breakthrough in removing these metals. For Zn, Cd and Ni, this breakthrough occurred even without HA being added. Adding kaolinite (representative of SS) had no effect on Pb and Cu, but it did on the other metals.

## 1. Introduction

Heavy metals, dissolved organic carbon (DOC), and suspended solids (SS) are serious pollutants of water. Of these, heavy metals are generating a great deal of concern due to their acute toxicity and long-term accumulation and persistence. Heavy metals at high concentrations may cause neuro-behavioural disorders, mental retardation, various types of cancers, kidney damage, and can even cause death when humans are exposed to them in high concentrations [1]. They can also adversely affect aquatic life in many ways [2].

Many countries have legislation and regulations governing the treatment of wastewater before it enters water bodies [3]. There are many treatment practices for removing pollutants from wastewater. Of these, bed filtration using adsorbents has been widely employed as an effective treatment strategy for removing organic and inorganic pollutants from storm water [2,4]. Granular activated carbon (GAC) is a widely used and versatile adsorbent in the water treatment process because it can remove many pollutants, such as organic carbon, turbidity, and nutrients [5,6,7,8]. GAC has also proved to be effective in removing many heavy metals [2,9,10]. The efficiency of GAC in removing pollutants using the adsorption process is due to its large surface area (500–1500 m^2^/g), internal microporosity, the presence of different types of functional groups, low cost, and easy availability [6,11,12]. Though GAC can remove heavy metals, its removal efficiency may depend on the concentrations of other pollutants, such as SS and DOC in the water. These effects, however, have been seldom investigated [2].

A major component of naturally occurring DOC is humic acid (HA), as it can represent up to 90% of DOC [2,13]. Humic acid has excellent binding properties and electrostatic interactions with heavy metals, leading to metal-organic complexes. These complexes can affect the adsorption of heavy metals on GAC. Heavy metal adsorption in the presence of HA may depend on several factors, such as surface charge of the adsorbent and type and abundance of functional groups in HA, concentration of HA, *etc*. In addition to GAC, SS can also adsorb heavy metals and HA and their complexes. Therefore, studies on the adsorptive removal of pollutants from water need to consider all these factors simultaneously and not in isolation. The novelty of the current study is the simultaneous consideration of these factors during the removal of heavy metals by adsorption in fixed-bed columns, especially their interactions that were not previously reported. Evaluating the removal mechanism using zeta potential data and chemistry of adsorption and metal complexation with HA is an additional innovation of this study.

The aims of this study were: firstly, to determine the effect of DOC and SS (a measure of turbidity) on the removal of heavy metals (Cu, Zn, Pb, Cd and Ni) from storm water using GAC fixed-bed columns; and secondly, to investigate their interactions during the removal process. The reason for studying Cu, Zn, Pb, Cd, and Ni was that these metals are widespread environmental pollutants that have aroused serious concerns. They can exist at high concentrations in urban and industrial storm water throughout the world [14,15]. GAC was mainly used as an adsorbent medium in this study because this is a common adsorbent used to remove many pollutants of water. HA was utilised as a representative for naturally occurring DOC [16,17] and kaolinite as a representative of SS or turbidity [2,13]. 

## 2. Experimental Methodology

### 2.1. GAC 

Granular activated carbon was purchased from James Cummins P/L, Australia. It had a nominal size of 0.3–2.4 mm and BET surface area of 750 m^2^/g [18]. A zetasizer nano instrument (Nano ZS Zne 3600, Malvern, UK) served to measure the zeta potential of GAC suspensions. The mean values of triplicate measurements made by the instrument were used in this study. Zeta potential was measured after adjusting the pH of GAC suspensions in deionised water (0.1 g/L) to pH 3–10 and equilibrating for 6 h. 

### 2.2. Humic Acid, Kaolinite and Chemicals

Humic acid and kaolinite were obtained from Sigma-Aldrich (USA). The approximate molecular weight of HA was 1000 g/mol (as determined by LC-OCD analysis). Analar grade nitrate salts of heavy metals (Cu, Zn, Pb, Cd, Ni) were employed in the study. These salts were also obtained from Sigma-Aldrich (USA). Zeta potential of kaolinite was determined by a method similar to that used for GAC. 

### 2.3. Batch Experiments

The heavy metals’ adsorption characteristics were determined using batch experiments. The experiments had initial metal concentrations of 5 mg/L and GAC doses of 0.1 to 7.5 g/L. Similar experiments were also conducted on the adsorption of heavy metals in the presence of HA at a concentration of 10 mg/L. Initial pH of the suspensions was maintained at 6.5 ± 0.1 to simulate pH to approximately the same as that of Australian storm waters, which is 6.68–7.28 [19]. The adsorption experiments were carried out in a flat shaker using 200 mL Pyrex vessels containing 100 ml heavy metal solutions and a specific dose of adsorbent, and then shaken at 120 rpm for 24 h at 24 ± 1 °C. The background ionic strength was retained at 0.001 M NaNO_3_. After 6 h of agitation the pH was adjusted back to the initial pH of 6.5 using 0.1 M NaOH or 0.1 M HNO_3_, in order to eliminate the possibility of any major pH changes during adsorption [2,20]. After 24 h of agitation the suspensions were filtered using filter disks with 0.45 µm openings. Heavy metals concentrations in the filtrate were analysed using a Microwave Plasma-Atomic Emission Spectrometer (Agilent 4100 *MP-AES).* The amount of adsorbed heavy metals at equilibrium was calculated using Equation (1):

(1) $$q_{e}=\;\frac{\left(C_{o}-\;C_{e}\right)\;V}{M}$$

where, *C_0_* = initial concentration of heavy metal (mg/L); *C_e_* = equilibrium concentration of heavy metal (mg/L); V = volume of solution (L); and *M* = mass of GAC (g). Prior to the adsorption studies the metal solutions were passed through the filter disks and it was found that no metals were adsorbed to the filter disks.

### 2.4. Fixed-Bed Column Experiments

Initially, the GAC was washed thoroughly with deionised water in a basin to remove any fine and floating particles. The washed GAC was packed within an acrylic fibre column (2-cm internal diameter) to a height of 90 cm. The amount of GAC used was 100 g. Deionised water was passed upwards through the column at a velocity of 5 m/h for 5 min to expel the air in the particle pores. Experiments were then conducted by filtration of tap water spiked with Zn, Pb, Cu, Ni, and Cd at the same velocity in the downward direction using peristaltic pumps. The solution’s flow rate was 26.4 mL/min. One pump was installed before the water entered the column and the other when the water left the column. The empty bed contact time (EBCT) at this filtration velocity was 10.8 min. The concentrations of Zn, Pb, Cu, Ni, and Cd in the influent water were 2.0, 1.0, 0.6, 0.06, 0.04 mg/L, respectively. Higher concentrations of heavy metals than those commonly found in Australian storm waters [21] were used to simulate the high concentrations normally observed in the first flush after long dry periods [22,23] and in industrial spills. Australian guideline limits for Cd, Cu, Pb, Ni, and Zn in water for long-term irrigation use are 0.01, 0.5, 2, 0.2, and 2 mg/L, respectively [24]. Their corresponding values for marine water ecosystems are 0.0014, 0.003, 0.0066, 0.2, and 0.023, respectively [24]. Samples were collected at 10 min, 30 min, and thereafter every hour and analysed for heavy metals, DOC and turbidity. A Hach Model 2100P Turbidimeter measured the turbidity levels followed by the samples that were filtered through 0.45 µm disks. DOC was measured in the filtrate using Multi N/C 2000 analyser (Analytik Jena AG). Heavy metals concentrations in the filtrate were measured using a Microwave Plasma-Atomic Emission Spectrometer (Agilent 4100 *MP-AES).*

Ding the experiments on the effect of DOC and SS on GAC’s removal of heavy metals, tap water spiked with heavy metals with and without the addition of HA (representing DOC) and kaolinite (representing SS) was utilised. HA and kaolinite, each with an initial concentration of 10 mg/L, was used because this concentration produced a DOC concentration of 5 mg/L and turbidity level of 12 NTU. These were approximately the same as those found in the storm water collected from Carlton, Sydney. Filtration experiments were run continuously for 80 h and effluent samples were collected more frequently at the initial stages and less frequently after 24 h. DOC, pH, turbidity and heavy metals were measured in these samples.

## 3. Results and Discussion

### 3.1. Zeta Potential 

**Figure 1:**
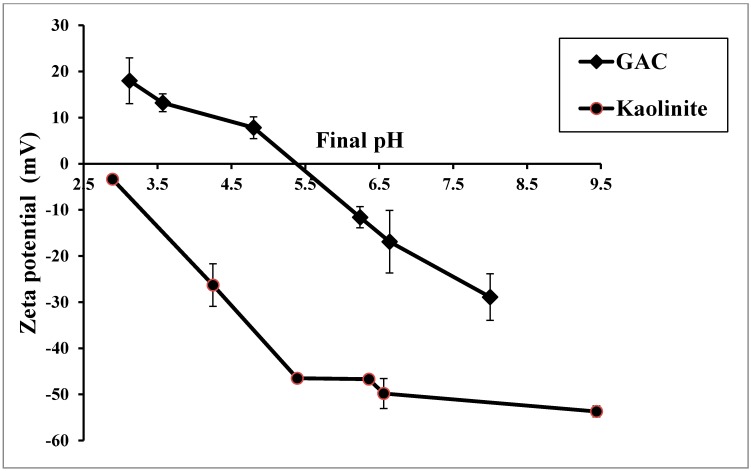
Mean zeta potential values of GAC and kaolinite at different pH values in 0.001 M NaNO_3_ after 6 h of agitation with standard deviations (GAC and kaolinite doses were 0.1 g/L and 0.2 g/L, respectively).

Zeta potential of GAC decreased when pH increased. The pH at which the zeta potential is zero is called the zero point of charge (ZPC). Figure 1 shows that the ZPC of GAC tested was 5.3. Faust and Aly [25] reported a ZPC range between 4.75 and 7.10 for five types of activated carbons tested. At pH less than the ZPC, GAC had a net positive surface charge and at pH above the ZPC, it had a net negative charge. GAC had a net negative charge at the pH of 6.5 used in the experiment, indicating that GAC had a high affinity for the positively charged heavy metals. The effect of pH on the kaolinite’s zeta potential was similar to that of GAC but the ZPC of kaolinite was much lower (pH 2.8) (Figure 1). Zeta potential of kaolinite was more negative than that of GAC at all pH levels. The difference in the zeta potentials ranged from 20 mV to 50 mV and at pH 6.5, where the heavy metals adsorption studies were conducted it was 35 mV.

### 3.2. Heavy Metals Adsorption by GAC in Batch Experiments 

The adsorption of heavy metals by GAC from solutions in the absence and presence of HA was conducted at pH 6.5. The adsorption data fitted satisfactorily to the Langmuir adsorption isotherm model described below [26] (Table 1).

(2) $$Q_{e}=\frac{q_{max}K_{L}C_{e}}{1+K_{L}C_{e}}$$

where *q_max_* = the maximum amount of the heavy metal adsorbed per unit weight of the GAC (mg/g) and K_L_ = Langmuir adsorption constant (L/mg). 

This model can be linearised as follows:

(3) $$\frac{C_{e}}{Q_{e}}=\frac{1}{q_{max}K_{L}}+\frac{C_{e}}{q_{max}}$$

From the inverse of the slope of the graph of *C_e_/Q_e_* *vs* *C_e_*, q_max_ was calculated.

The Langmuir adsorption maximum for GAC in the absence of HA decreased in the following order: Pb, Cu > Zn > Ni, Cd (Table 1). This order is similar to that of the solubility product of the metal hydroxide (M(OH)_2_) precipitate (pK_sp_ for Pb, Cu, Zn, Ni, and Cd hydroxides). These were 19.9, 19.3, 16.5, 15.2, 14.4, respectively [27,28]. The adsorption maxima also followed the reverse order of the metals’ first hydrolysis constant (MOH^+^ formation) (pK_1_ of hydroxyl complexes of Pb, Cu, Zn, Ni, and Cd are 7.7, 7.7, 9.0, 9.9, and 10.1, respectively [29]). The higher the pK_sp_ the more likely metal hydroxides precipitation would occur, whereas a lower pK_1_ value made it easier for the metal to produce the soluble hydroxyl complex. The higher adsorption capacity of the metals having higher pK_sp_ values is due to these metals forming precipitation on the adsorbent surface [30]. Precipitation on the adsorbent surface can occur at pH levels less than those at which precipitation occurs in solution. This is due to the adsorbent providing a nucleus promoting precipitation. The metals, which easily form hydroxyl complexes (lower pK_1_ values, e.g., Pb and Cu), had larger adsorption capacities because these complexes have higher affinity for adsorption than divalent metal ions.

**Table 1:** Langmuir maximum adsorption capacity (*q_max_* mg/g), coefficients of determination of Langmuir isotherm fit to data (R^2^) and adsorption capacity at an equilibrium metal concentrations equivalent to the influent concentrations (Zn 1.80, Cu 0.99, Cd 0.11, Ni 0.12, Pb 1.03) used in column study (*q_e_* mg/g) for heavy metal adsorption on GAC at pH 6.5.

Metals	GAC					GAC + HA				
	*q_max_* mg/g	K_L_ L/mg	R^2^	n	*q_e_* mg/g	*q_max_* mg/g	K_L_ L/mg	R^2^	n	*q_e_* mg/g
Cu	11.8	42.0	0.99	6	11.0	3.7	67.2	0.99	6	3.5
Pb	11.9	1.8	0.91	6	8.1	7.5	1.7	0.75	7	4.0
Zn	3.3	6.1	0.94	5	2.8	3.2	5.7	0.96	7	2.6
Ni	1.8	15.3	0.98	6	1.3	2.5	19.9	0.98	6	2.0
Cd	2.0	4.4	0.95	7	0.9	1.6	8.7	0.99	6	0.9

n = No. of data points.

In the presence of HA the Langmuir adsorption capacity of Cu and Pb drastically decreased (Table 1). The reason for this is probably that these two metals form strong complexes with HA in solution, thus they are not precipitated on the GAC surface. Sholkovitz and Copland [31] also found that adding fulvic acid to clay suspensions containing metal ions at high pH values (>6.5) prevented Pb, Cu, and Cd from forming hydroxide precipitates. Pandey *et al.* [32] reported that the stability constants for the metal-HA complex followed the order, Cu > Pb > Ni > Cd, Zn. HA exerted little effect on the adsorption capacity of Zn, Ni, and Cd because they have low stability constants. 

### 3.3. Heavy Metals Removal in Column Experiments in the Absence of HA and Kaolinite 

**Figure 2:**
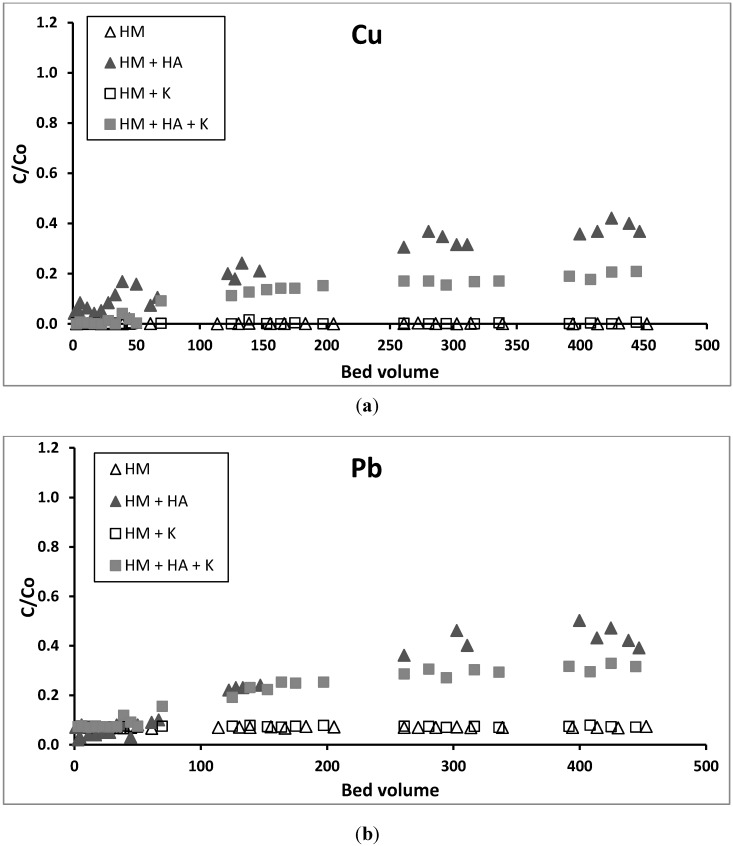
Breakthrough plots of (**a**) Cu and (**b**) Pb in the column study (HM—heavy metals, HA—humic acid, K—kaolinite).

**Figure 3:**
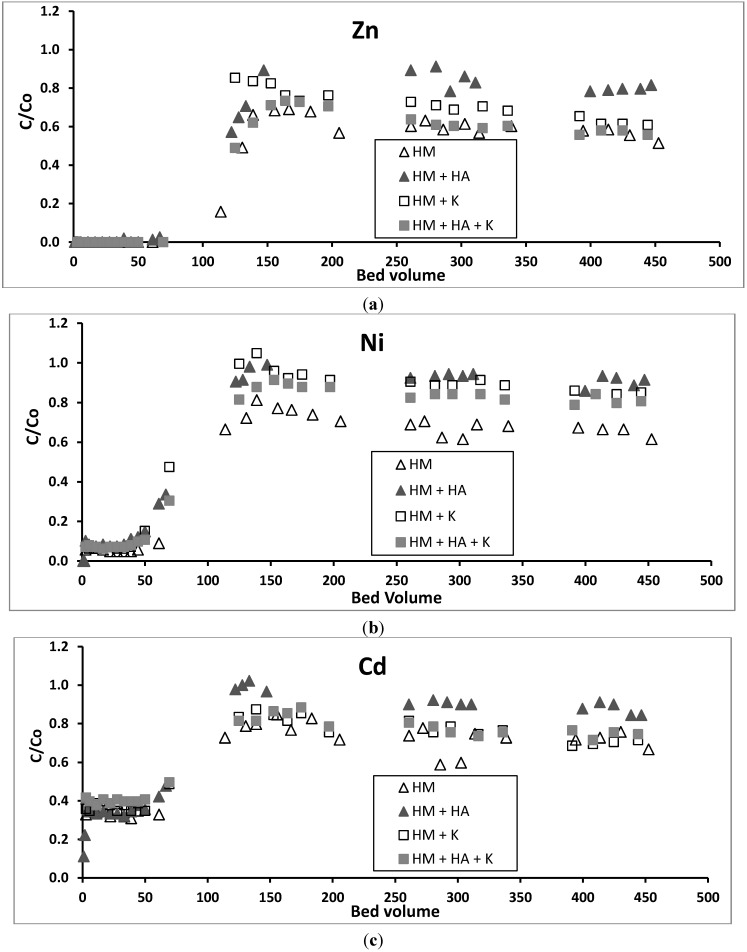
Breakthrough plots of (**a**) Zn, (**b**) Ni, and (**c**) Cd in the column study (HM—heavy metals, HA—humic acid, K—kaolinite).

**Figure 4:**
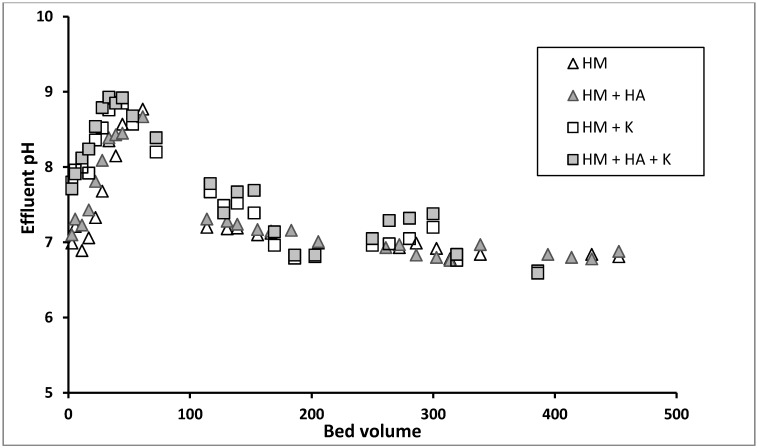
Effluent pH variation with time in the column study (HM—heavy metals, HA—humic acid, K—kaolinite).

Column experimental results showed that no breakthrough was achieved for Cu and Pb up to 450 bed volumes (BV) (Figure 2). However, for Cd, Zn and Ni a breakthrough began at 50 BV and was nearly complete when 130 BV was reached (Figure 3). The absence of a breakthrough for Cu and Pb is probably because these metals have formed surface precipitation on GAC at the pH of 6.5 used in the influent in addition to forming Cu(OH)^+^ and Pb(OH)^+^, which had a stronger adsorption tendency than Cu^2+^ and Pb^2+^ onto GAC, unlike the other metals as discussed previously under batch experiments. Almost all Zn would have precipitated up to 50 BV because of the solution’s elevated pH in the adsorbent during this period (Figure 4). Other studies have also reported this initial rise in pH followed by a decrease reaching the pH of the influent pH in fixed-column adsorption studies [33,34]. The increase in the pH was explained as being due to initial adsorption of hydrogen ions from the solution and dissolution of basic impurities from the GAC [33]. A large proportion of Ni and Cd may have also precipitated or been adsorbed, resulting in the low breakthrough of these metals during the first 50 BV. These metals have lower K_sp_ and lower concentrations than Zn and therefore might have not completely precipitated or formed hydroxylated species. 

Table 2 shows the cumulative percentage removal of heavy metals after 80 h (450 BV). Heavy metals adsorption in the column followed the order Cu, Pb > Zn > Cd, Ni, which is similar to the batch adsorption results highlighted in Table 1. In addition to the explanation presented for this trend in the batch studies, the influent concentration differences in the influent solution can also explain the adsorption order. Ni and Cd adsorption was low compared to the other metals because of: firstly, the relatively low concentrations of these metals in the influent water; and secondly, the low adsorption capacities of these metals inferred from batch adsorption results.

**Table 2:** Effects of humic acid (HA) and kaolinite (K) on the percentages * of cumulative removals of heavy metals (HM) by GAC (Influent concentrations (mg/L): Zn 1.80, Cu 0.99, Cd 0.11, Ni 0.12, Pb 1.03).

	Zn	Cu	Cd	Ni	Pb
HM only	53.7	99.9	36.7	39.9	93.0
HM + HA	29.4	72.1	15.2	17.6	59.8
HM + HA + K	48.6	84.3	26.6	24.8	70.5
HM + K	39.2	98.0	28.1	18.1	86.7

* Percentage of cumulative metal removal = (cumulative metal added—cumulative metal in effluent)/cumulative metal added * 100.

### 3.4. Effect of HA on Removal of Heavy Metals in Column Experiments

**Figure 5:**
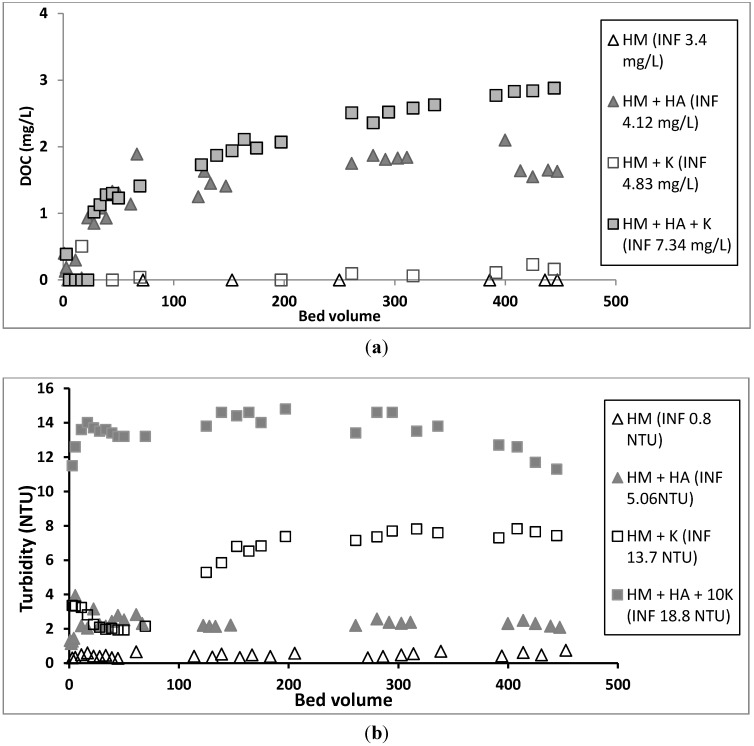

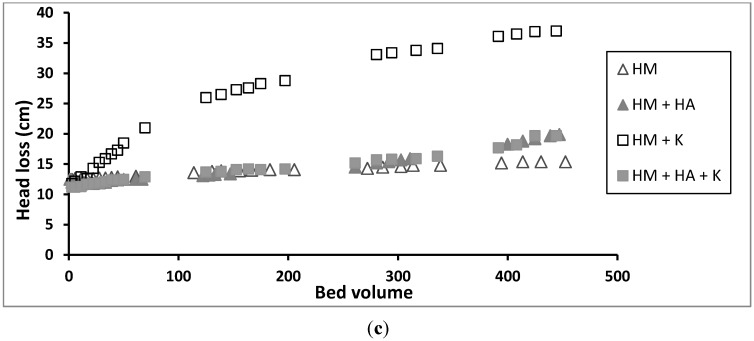
(**a**) DOC, (**b**) turbidity removals and (**c**) head loss generation in the column study (HM—heavy metals, K—kaolinite, INF—influent).

The addition of HA to the metal solution significantly reduced all heavy metals adsorption on GAC (Table 2, Figure 2 and Figure 3) with the highest reduction being that of Cu and Pb. This is because the heavy metals formed heavy metal-HA complexes in the solution, which prevented the metals from adsorbing or precipitating on GAC surfaces. Thus, a significant percentage of all heavy metals transported with HA into the effluent. A significant amount of HA movement into the effluent is supported by the decline in DOC from the influent to the effluent (Figure 5).

Batch adsorption of the metals at an equilibrium metal concentration equivalent to the influent concentrations used in the column study (q_e_ mg/g) revealed that there was a marked reduction of adsorption of Cu and Pb in the presence of HA (Table 1). This was observed in the column study, but had little effect on the adsorption of other metals unlike the column study results. The difference in the results between the static batch and dynamic column studies is explained by the fact that equilibrium between the solution phase metals and adsorbed phase metals had not been reached in the column study. This was due to the short residence time of metals in the column, especially at the high flow rate used, unlike in the batch study [35]. The adsorption capacities for Cu, Pb, Zn, Ni, and Cd in the column study were 1.25, 1.21, 1.22, 0.06, and 0.05 mg/g, respectively. In the presence of HA, these values fell to 0.90, 0.78, 0.67, 0.03, and 0.02 mg/g, also respectively. These values are lower than the values obtained in the batch study for solution metal concentrations same as those in the column study (Table 1). However, the column study is closer to the operational conditions in the real full-scale treatment system and consequently the results from this study are more applicable to real practical conditions.

### 3.5. Effect of Kaolinite on Removal of Heavy Metals in Column Experiments

Adding kaolinite to the heavy metals solution significantly reduced the adsorption of Zn, Cd and Ni on GAC (Table 2, Figure 3). This is probably due to these positively charged metals adsorbing onto the negatively charged surfaces of the finer sized kaolinite by Coulombic forces (Figure 1) and passing through the column along with the kaolinite to the effluent. High turbidity in the effluent of the column (Figure 5) confirms that significant amounts of kaolinite passed through the column into the effluent. The reason for these metals adsorbing more on kaolinite than on GAC is due to kaolinite having much a higher negative zeta potential and therefore higher number of negative charges than GAC (Figure 1). In contrast to Zn, Cd, and Ni, the adsorption of Pb and Cu was not significantly affected by the presence of kaolinite (Table 2, Figure 2). This may be due to the high removal of Pb and Cu by surface precipitation onto GAC and kaolinite. Pb and Cu precipitated on the surface of kaolinite were probably retained in the column pores and this affected the flow of the solution. The large head loss observed in the column when kaolinite was present (Figure 5) is due to this precipitation and blockage of the pores. 

### 3.6. Effect of HA and Kaolinite together on Removal of Heavy Metals in Column Experiments

The addition of both HA and kaolinite to the metals solution reduced the heavy metals adsorption compared to that from the metals solution alone. However, the reduction in adsorption was less than when only HA was added to the metals solution (Table 2, Figure 2 and Figure 3). This is probably because part of the HA added was adsorbed to the kaolinite particles and retained in the column, leaving a smaller amount of HA to complex with heavy metals and move down the column into the effluent. Although both HA and kaolinite have net negative charges at pH 6.5 as used in the experiment, some of the HA could have adsorbed to the positive charges on the edges of the kaolinite [36].

## 4. Conclusions

Fixed-bed column containing GAC effectively removed heavy metals from water. At a pH level close to that of storm waters (*i.e.*, pH 6.5) the removal of heavy metals by the GAC column decreased in the order of Cu, Pb > Zn > Cd, Ni. This outcome was consistent with the metals adsorption capacity when assessed by Langmuir adsorption isotherm.

The presence of DOC and turbidity (SS) in storm water of pH 6.5, as simulated by spiking aqueous solutions with HA and kaolinite, respectively, exerted different degrees of influence on the removal of heavy metals. Adding HA reduced the efficiency in removing Cu and Pb very strongly, but the other metals only marginally. Kaolinite addition had no effect on the removal of Pb and Cu but it reduced the removal of the other metals. Kaolinite and HA together reduced the removal of all metals but the effect was less than that when only HA was added. Overall, the study demonstrated the strong influence of HA and kaolinite additions on the removal of heavy metals from storm water by GAC, which varied according to the type of heavy metal.
